# Combined lifestyle, mental health, and mortality in US cancer survivors: a national cohort study

**DOI:** 10.1186/s12967-022-03584-4

**Published:** 2022-08-19

**Authors:** Zhao-yan Liu, Chen Wang, Yao-jun Zhang, Hui-lian Zhu

**Affiliations:** 1 Department of Nutrition, School of Public Health, Sun Yat-Sen University, 74 Zhong Shan Road 2, Guangzhou, 510080 Guangdong China; 2 Guangdong Provincial Key Laboratory of Food, Nutrition and Health, School of Public Health, Sun Yat-Sen University, Guangzhou, Guangdong China; 3 Department of Hepatobiliary Oncology, Sun Yat-Sen University Cancer Center, 651 Dongfeng Road East, Guangzhou, 510060 Guangdong China; 4 State Key Laboratory of Oncology in South China, Sun Yat-Sen University Cancer Center, Guangzhou, Guangdong China

**Keywords:** Lifestyle, Mental health, Mortality, Cancer survivor

## Abstract

**Background:**

Adopting healthy lifestyles and staying mentally health are two cost-effective modifiable strategies that cancer survivors can implement in self-management. We aimed to evaluate the independent, mediation, interaction, and joint associations of combined lifestyle and mental health with mortality in cancer survivors.

**Methods:**

We performed a cohort study including 3145 cancer survivors from National Health and Nutrition Examination Survey (2005–2018). A healthy lifestyle score was constructed based on post-diagnosis body mass index, physical activity, diet, smoking, and drinking. Post-diagnosis mental health was assessed by Patient Health Questionnaire (PHQ-9). Hazard ratios (HRs) and 95% confidence intervals (CIs) for all-cause, cancer, and non-cancer mortality were computed using Cox proportional hazards regression models.

**Results:**

After 20,900 person-years of follow-up (median, 6.3 years), cancer survivors with higher lifestyle score had decreased mortality, independent of mental health. Compared to participants with lower lifestyle score (0–1), HRs (95% CIs) for all-cause and non-cancer mortality among those with higher lifestyle score (3–5) were 0.68 (0.52–0.89) and 0.69 (0.56–0.85), respectively. 6.2–10.3% of the associations were mediated by mental health. Similar trends were observed among participants categorized by mental health, those with better mental health had lower mortality, independent of lifestyle. Participants with better mental health benefited more from adopting healthy lifestyles, and vice versa. Combinations of higher healthy lifestyle score and better mental health were associated with significant decreased mortality, the lowest mortality was seen in participants with highest healthy lifestyle score and concurrently with best mental health.

**Conclusions:**

For the first time, in this cohort study with a nationally representative sample of US cancer survivors, we comprehensively explored the complex associations of lifestyle, mental health, and mortality. Evidence derived from this study may give much confidence to cancer survivors and healthcare providers that, changing one’s lifestyle and/or staying mentally healthy after cancer diagnosis can improve survival.

**Supplementary Information:**

The online version contains supplementary material available at 10.1186/s12967-022-03584-4.

## Background

Cancer is the second leading cause of death in US [1]. Coupled with the advances in early detection, treatment, and accelerating pace of population ageing, the number of cancer survivors is rapidly growing [1]. It was estimated that there will be approximately 1.9 million new cancer cases diagnosed in US in 2022 [1], equivalent to over 5000 new cases each day. By 2030, the number is projected to increase to 22.1 million [2]. After the diagnosis of cancer, treatment received from professional health care providers and self-management conducted by the patients are the most important determinants of survival. However, during the coronavirus disease 2019 (COVID-19) pandemic period, the treatment of cancer was frequently delayed, interrupted or even cancelled because of health care setting closures and fear of COVID-19 exposure [1]. Thus, it is urgent to identify cost-effective modifiable strategies that cancer survivors can easily implement in self-management to improve their survival.

Mounting evidence has revealed that adopting healthy lifestyles is one of the “best buy” strategies for cancer management [3]. Earlier studies mostly focused on the associations of individual lifestyle with cancer mortality [4–9]. In the past decade, numerous researchers have emphasized the importance of adopting an overall healthy lifestyle for cancer management, instead of focusing on individual factors. Since 2012, after a combined healthy lifestyle score was constructed according to the cancer prevention recommendations of the World Cancer Research Fund/American Institute for Cancer Research (WCRF/AICR), a large number of studies have explored the associations of this score with health outcomes in cancer survivors [3, 10–18]. However, important gaps remain. First, in the WCRF/AICR score, tobacco smoking was not included as a component [3, 10–18], which might underestimate the association of the score with outcomes, since lung cancer remains the leading cause of cancer death in US [1], and avoiding smoking is the most well-established lifestyle suggested for lung cancer survivors. Second, when assessing the overall diet quality, instead of using the most widely used healthy eating index (HEI) in US, the WCRF/AICR score only included certain food groups, thus several other important food groups or nutrients that may affect outcomes were not comprehensively considered. Third, most of the aforementioned studies explored the associations of adherence to the lifestyle factors before diagnosis with health-related quality of life among cancer survivors, evidence linking long-term mortality is still scarce. Besides, lifestyle behaviors may significantly change after diagnosis of cancer, thus it is necessary to estimate the impact of lifestyle factors post-diagnosis other than pre-diagnosis.

Mental health is another important factor that has been suggested to explain the variations in mortality among cancer survivors [19]. Depression is a common complication that occurred in about 20–25% of cancer survivors [19, 20]. Growing studies accumulated to explore the associations of mental health with mortality among patients with lung cancer [21], prostate cancer [22], esophageal cancer [23], neuroendocrine neoplasms [24, 25], and breast cancer [26–28], yet the findings are still inconsistent, more evidences are warranted regarding to this setting. Moreover, at the behavioral level, mental health and healthy lifestyle can influence and reinforce each other. On one hand, patients with a depression status were reported to be less likely to adopt healthy lifestyle, including being less physically active, consuming more alcohol, having poor diet, and failing to maintain healthy body weight [29]. On the other hand, unhealthy lifestyle can also have negative impact on mental health, thus mental health is viewed as a mediator between healthy lifestyle and health outcomes [30]. However, it is not clear whether there is a mediation and joint association of healthy lifestyle and mental health on mortality among cancer survivors.

In the present study, we aimed to evaluate the independent, mediation, interaction, and joint associations of combined healthy lifestyles and mental health with mortality among a US nationally representative sample of cancer survivors. We hypothesized that cancer survivors with more healthy lifestyle behaviors or better mental health had significantly decreased mortality, independent of each other. Meanwhile, participants adherence to healthy lifestyle and concurrently staying mentally healthy would benefit more compared to their counterparts.

## Methods

### Study population

This cohort study used data extracted from the 2005–2018 cycles of National Health and Nutrition Examination Survey (NHANES), which is a periodic, nationally representative sampling survey conducted by the National Center for Health Statistics of the Centers for Disease Control and Prevention. Data on cancer diagnosis were self-reported. First, participants were asked, “Have you ever been told by a doctor or other health professional that you had cancer or a malignancy of any kind?” Participants were defined as cancer survivors if they responded yes. Then, cancer types and age at each diagnosis were further asked, by “What kind of cancer was it?” and “How old were you when this cancer was first diagnosed?” After excluding participants who were pregnant, had missing information on lifestyle, mental health, and mortality status, 3145 cancer survivors retained (Additional file 1: Figure S1).

### Assessment of lifestyle

We selected 5 lifestyle factors to construct a healthy lifestyle score, based on previously published studies as well as the WHO recommendations for the prevention and control of noncommunicable diseases [31–33], including diet, physical activity (PA), smoking, alcohol consumption, and body mass index (BMI). For each lifestyle, participants scored 1 point if they were classified as achieving a healthy level, otherwise they scored 0 point. Then the combined healthy lifestyle score was calculated by summing up the 5 scores with a range from 0 to 5, higher scores reflected adopting healthier lifestyle.

Data on diet intake was collected for two nonconsecutive days by the 24-h dietary recall method (Day1 and Day2). Consumptions of food groups and nutrients were estimated using the US Department of Agriculture (USDA) Nutrient Database for Dietary Studies and Food Patterns Equivalents Database. In the present study, for most participants (92.3%) who had data on both Day1 and Day2, the mean values were used. However, only values of Day1 were used if participants lacked Day2 data. We calculated the HEI-2015 to reflect the overall diet quality, according to the 2015–2020 Dietary Guidelines for Americans (DGA) [34], based on the MyPyramid Equivalents Database 2.0 for USDA Survey Foods (Additional file 1: Table S1). Participants with a HEI-2015 score in the top 40% [31, 34] of this study were classified as achieving a healthy diet quality and were assigned 1 point.

Information regarding PA, smoking, and alcohol consumption were obtained through structured questionnaire. For PA, questions related to daily activities (DA, including activities from work and transportation) and leisure time physical activities (LTPA) were asked, then weekly metabolic equivalent hours (MET.hours/week) of DA and LTPA were calculated. To harmonize with previous studies [3, 10–18], we used LTPA to reflect the level of PA in the main analyses. Participants who had reported any level of LTPA were considered as having a healthy level of PA and were assigned 1 point. Participants were asked “Have you smoked at least 100 cigarettes in your entire life?” and were defined as non-smokers if they responded no. For those responded yes, they were further asked “Do you now smoke cigarettes?” participants were defined as current smokers if they responded yes, and former smokers if they responded no. Non-smokers were considered as having a healthy level of smoking, and were assigned 1 point, both current and former smokers were assigned 0 point. Questions focused on lifetime and current alcohol consumption (past 12 months) were asked, non-drinkers were participants who reported consuming less than 12 alcohol drinks each year, low-to moderate drinkers were defined as < 14 drinks/week for men or < 7 drinks/week for women, heavy drinkers were defined as ≥ 14 drinks/week for men or ≥ 7 drinks/week for women. According to the 2015–2020 DGA [34], low-to moderate drinkers were considered as having a healthy level of alcohol consumption and were assigned 1 point, both non-drinkers and heavy drinkers were assigned 0 point. Weight and standing height were measured. BMI was calculated as weight in kilograms divided by height in meters squared. Participants with a BMI of 18.5–24.9 (kg/m^2^) were considered as having a healthy body shape and were assigned 1 point.

### Assessment of mental health

Since the cycle of 2005–2006 in NHANES, mental health was assessed by a 9-item depression screening instrument, the Patient Health Questionnaire (PHQ-9). The frequency of depression symptoms over the past 2 weeks was administered. For each item, a point of 0–3 was given to the response categories "not at all," "several days," "more than half the days," and "nearly every day", respectively. Then all points were summed up to a total score of 0–27. The cut-off of 5–9 and ≥ 10 were used to define the presence of mild and major depression [35], respectively.

### Ascertainment of mortality

Data for deaths were obtained by linking to the NHANES-linked National Death Index public access files. Cause of death was defined using the International Statistical Classification of Disease, Tenth Revision (*ICD-10*). All-cause, cancer, and non-cancer mortality were primary outcomes. Death from all reasons was defined as all-cause mortality, cancer mortality was defined as *ICD-10* codes C00-C97, otherwise, deaths were defined as non-cancer mortality. Follow-up time was calculated from the date of interview to the date of death, or the end of follow-up (December 31, 2019), whichever came first.

### Definition of covariates

Basic demographic data, age at interview (continuous), sex (men, women), education (less than high school, high school or equivalent, college or above), ratio of family income to poverty (RFIP, < 1.3, 1.3–3.5, > 3.5), race and ethnicity (Mexican American, non-Hispanic white, non-Hispanic black, others) were included. In addition to the above-mentioned 5 lifestyle factors, data on nighttime sleep duration was also included. Moreover, several medical condition-related data were assessed and categorized based on self-report, laboratory measurements, and examination. Prevalent diabetes was defined by a self-reported diagnosis, or currently taking insulin or prescription drugs to treat diabetes, or had a fasting plasma glucose ≥ 7.0 mmol/L, or a postprandial 2-h plasma glucose ≥ 200 mg/dL, or a glycated hemoglobin A_1c_ ≥ 6.5%. Prevalent hypertension was defined by a self-reported diagnosis, or currently taking anti-hypertensive drugs, or had a systolic/diastolic blood pressure ≥ 140/90 mmHg. Prevalent dyslipidemia was defined by a self-reported diagnosis, or currently using prescription drugs for lipid-modifying, or had a total cholesterol ≥ 200 mg/dL, or triglyceride ≥ 150 mg/dL, or LDL-cholesterol ≥ 130 mg/dL, or HDL-cholesterol < 40 mg/dL for men, HDL-cholesterol < 50 mg/dL for women. History of cardiovascular disease (CVD) was defined as a self-reported diagnosis of any of the following disease, including stroke, angina, heart attack, coronary heart disease, or congestive heart failure.

### Statistical analyses

According to NHANES analytic guidelines, all analyses in this study incorporated sample weights, clustering, and stratification, to estimate appropriate variance and ensure nationally representative of US cancer survivors. Baseline characteristics were described across different levels of healthy lifestyle score (0–1, 2, 3–5) and mental health (PHQ-9 score 0–4, 5–9, ≥ 10), respectively. Data were presented as mean ± standard error (SE) for continuous variables and percentage for categorical variables. The differences of baseline characteristics were compared across the three groups by Rao-Scott Chi-squared test for categorical variables, and by general linear models for continuous variables, respectively.

Multivariable Cox proportional hazards regression models were used to calculate hazard ratios (HRs) and 95% confidence intervals (CIs) for the associations of healthy lifestyle score and mental health with mortality, independently. The proportional hazards assumption was satisfied by creating a product term of follow-up time and healthy lifestyle score, or a product term of follow-up time and PHQ-9 score in the models. Three multivariable models were eventually evaluated. Model 1 was adjusted for age at the time of interview, sex, education, RFIP, race and ethnicity, sleep duration, prevalent diabetes, hypertension, dyslipidemia, and history of CVD. Model 2 was additionally adjusted for the number of cancer types and age at the first cancer diagnosis. Moreover, model 3 was mutually adjusted for PHQ-9 score or healthy lifestyle score, in regard to the association of healthy lifestyle score and mental health with mortality, respectively. In these analyses, the reference group was set as participants with unhealthy lifestyle (with a lifestyle score of 0–1), or as participants with poor mental health (with a PHQ-9 score ≥ 10). Mediation proportion was calculated by mental health (the mediator) for the association between healthy lifestyle score and mortality, using the difference method, by comparing estimates from models with and without the hypothesized mediator [34, 36].

Since significantly favorable associations were observed among higher healthy lifestyle score, all-cause and non-cancer mortality, also among better mental health, all-cause and non-cancer mortality, we further stratified the analyses by exploring whether adherence to healthy lifestyles was associated with protection against all-cause and non-cancer mortality in participants with different mental health status, and vice versa. A cross-product term of healthy lifestyle score (0–1, 2, 3–5) and mental health (PHQ-9 score 0–4, 5–9, ≥ 10) was included into the corresponding models to evaluate the multiplicative interactions. Additionally, to assess the joint associations, participants were reclassified into nine groups, by combination of mental health (PHQ-9 score ≥ 10, 5–9, 0–4) and healthy lifestyle score (0–1, 2, 3–5). Cox proportional hazards regression models were used to calculate HRs and 95% CIs adjusting for the same set of covariates in model 3, the group with lowest healthy lifestyle score (0–1) and poorest mental health status (PHQ-9 score ≥ 10) was set as the reference group. Meanwhile, the additive interaction effect between the healthy lifestyle score (0–1 point *vs.* 3–5 points) and mental health (PHQ-9 score ≥ 10 *vs.* PHQ-9 score = 0–4) was evaluated using the delta method, the relative excess risk due to interaction (RERI), attributable proportion (AP), synergy index (S), and the corresponding 95% CIs were calculated [37].

Finally, several sensitivity analyses were conducted. First, we excluded participants died within the first 2-year of follow-up, or those with missing covariates, then we repeated the analyses of independent associations with healthy lifestyle score and mental health on mortality. Second, we constructed a series of new lifestyle scores to reevaluate their associations with mortality. (1) The LTPA was replaced by DA, since over 80% of participants reported hardly any level of LTPA. Participants in the top third of DA distribution (MET.hours/week) were considered as having a healthy level of PA, otherwise were unhealthy. (2) Nighttime sleep duration was included into the score. Participants reported a sleep duration of 6–8 h/day were considered as having a healthy level of sleep duration, otherwise were unhealthy [38]. (3) A weighted healthy lifestyle score was reconstructed, based on β coefficients of each lifestyle assessed by the Cox regression model with all 5 lifestyles included. (4) We recoded 0–2 points to each lifestyle factor (Additional file 1: Table S2), then summed up the individual scores with a range of 0–10. Third, to evaluate the contribution of each individual lifestyle to mortality outcomes, we reconstructed several new healthy lifestyle scores by omitting 1 lifestyle each time (scaled 0–4), then participants were reclassified into scores of 0–1, 2, and 3–4.

All analyses were performed using the R software 4.0.5 (the “survey” package), the SPSS V.26.0 software (SPSS Inc., Chicago, IL, USA) and the GraphPad PRISM 8.0 (La Jolla, California). A two-sided *P*_-value_ < 0.05 was considered statistically significant.

## Results

### Baseline characteristics

Baseline characteristics are demonstrated in Table 1 and Table S3 (Additional file 1). Of the 3145 cancer survivors (weighted mean [SE] age, 62.7 [0.4] years; 43.1% male), most of them (89.5%) had one kind of cancer, 613 (24.0%) people were first diagnosed with cancer when they were younger than 40 years old. Many cancer survivors also suffered from a variety of other diseases, more than half of them had hypertension (58.6%) and dyslipidemia (78.9%). 822 (21.2%) were non-drinkers, 262 (10.1%) were heavy drinkers, more than half of them were former or current smoker (53.7%). 495 (15.5%) and 318 (8.5%) had mild and major depression, respectively. As expected, cancer survivors with higher healthy lifestyle score had better mental health. Several similar trends were observed among participants categorized by healthy lifestyle score or mental health. Compared with their counterparts, participants with a healthy lifestyle score of 3–5 (better lifestyle) or a PHQ-9 score of 0–4 (better mental health) were richer and higher educated, had better sleep status and better medical conditions. Particularly, poor mental health was observed among cancer survivors who were younger, and were first diagnosed with cancer when they were younger than 40 years old (Additional file 1: Table S3).

**Table 1:** Baseline characteristics^a^ of US cancer survivors according to healthy lifestyle score

Characteristics	Total	Healthy lifestyle score			
		0–1	2	3–5	*P*_*-value*_
PHQ-9 score^b^, No. (%)					
0–4	2332 (76.1)	857 (71.8)	814 (74.3)	661 (83.6)	< 0.001
5–9	495 (15.5)	190 (16.6)	201 (17.2)	104 (11.9)	
≥ 10	318 (8.5)	159 (11.6)	117 (8.5)	42 (4.5)	
HEI-2015^c^	55.1 (0.3)	48.0 (0.4)	55.1 (0.5)	64.1 (0.5)	< 0.001
LTPA^c^, MET.hours/week	5.7 (0.6)	0.6 (0.2)	3.3 (0.8)	15.2 (1.7)	< 0.001
Daily activity^c^, MET.hours/week	31.5 (1.7)	32.3 (2.9)	33.8 (3.1)	27.6 (2.5)	0.261
Alcohol drinking status, No. (%)					
Non-drinker	822 (21.2)	462 (32.6)	270 (19.4)	90 (9.2)	< 0.001
Low-to moderate drinker	2061 (68.7)	576 (49.9)	799 (73.3)	686 (86.5)	
Heavy drinker	262 (10.1)	168 (17.5)	63 (7.3)	31 (4.3)	
Smoking status, No. (%)					
Non-smoker	1419 (46.3)	236 (19.0)	580 (51.6)	603 (74.2)	< 0.001
Current smoker	486 (15.5)	299 (25.6)	148 (13.0)	39 (5.7)	
Former smoker	1240 (38.2)	671 (55.4)	404 (35.3)	165 (20.1)	
Quiting smoking ≥ 10 years, No. (%)	980 (30.2)	531 (10.0)	318 (6.0)	131 (3.2)	< 0.001
Sleep duration, hours/day					
6–8 h/day, No. (%)	2170 (71.3)	785 (67.7)	785 (71.5)	600 (75.4)	< 0.001
5–5.9 or 8.1–10 h/day, No. (%)	423 (14.8)	160 (14.5)	147 (13.8)	116 (16.5)	
< 5 or > 10 h/day, No. (%)	538 (13.9)	255 (17.8)	194 (14.7)	89 (8.1)	
Body mass index ^c^, kg/m^2^	29.1 (0.1)	31.4 (0.2)	29.4 (0.2)	25.8 (0.2)	< 0.001
< 18.5 kg/m^2^, No. (%)	47 (1.7)	29 (2.4)	13 (0.9)	5 (1.0)	< 0.001
18.5–24.9 kg/m^2^, No. (%)	790 (26.2)	63 (4.8)	283 (24.1)	444 (55.6)	
25.0–29.9 kg/m^2^, No. (%)	1109 (34.3)	507 (41.5)	406 (34.5)	196 (24.8)	
≥ 30.0 kg/m^2^, No. (%)	1199 (38.1)	607 (51.2)	430 (40.4)	162 (18.6)	
Age at interview ^c^, years	62.7 (0.4)	63.1 (0.5)	63.1 (0.5)	61.9 (0.7)	0.245
Gender, No. (%)					
Male	1492 (43.1)	566 (44.6)	548 (44.1)	378 (39.9)	0.279
Female	1653 (56.9)	640 (55.4)	584 (55.9)	429 (60.1)	
Ethnicity, No. (%)					
Non-Hispanic white	2145 (86.5)	812 (84.9)	761 (86.7)	572 (88.3)	0.013
Non-Hispanic black	451 (4.3)	198 (6.4)	162 (4.9)	91 (3.7)	
Mexican American	203 (2.4)	84 (2.8)	83 (2.8)	36 (1.5)	
Others	346 (5.9)	112 (5.8)	126 (5.6)	108 (6.6)	
Education level, No. (%)					
Less than high school	638 (11.9)	328 (17.7)	225 (10.9)	85 (6.1)	< 0.001
High school or equivalent	712 (21.4)	294 (24.5)	282 (25.2)	136 (12.7)	
College or above	1794 (66.7)	584 (57.8)	624 (63.9)	586 (81.2)	
Family income-to-poverty ratio, No. (%)					
< 1.3	656 (13.7)	317 (19.5)	236 (13.3)	103 (7.0)	< 0.001
1.3–3.5	1232 (37.3)	503 (42.1)	466 (39.1)	263 (29.0)	
> 3.5	1013 (49.0)	295 (38.3)	346 (47.6)	372 (64.0)	
Prevalent diabetes, No. (%)	871 (22.6)	399 (29.0)	321 (24.5)	151 (12.4)	< 0.001
Prevalent hypertension, No. (%)	2033 (58.6)	831 (64.0)	741 (61.4)	461 (48.3)	< 0.001
Prevalent dyslipidemia, No. (%)	2437 (78.9)	974 (81.9)	868 (78.8)	595 (75.2)	< 0.001
History of CVD, No. (%)	738 (18.7)	334 (22.8)	285 (20.2)	119 (11.6)	< 0.001
Number of cancer types, No. (%)					
1	2820 (89.5)	1087 (90.0)	1003 (88.5)	730 (90.0)	0.342
2	284 (9.3)	104 (8.8)	109 (9.6)	71 (9.4)	
≥ 3	39 (1.3)	15 (1.3)	19 (1.8)	5 (0.6)	
Age at cancer first diagnosed, years					
< 40 years, No. (%)	613 (24.0)	251 (24.5)	211 (23.7)	151 (23.8)	0.252
40–60 years, No. (%)	1249 (43.8)	454 (40.6)	470 (46.7)	325 (44.3)	
> 60 years, No. (%)	1283 (32.2)	501 (34.9)	451 (29.7)	331 (31.9)	

*PHQ* Patient Health Questionnaire, *CVD* cardiovascular disease, *HEI* healthy eating index, *LTPA* leisure time physical activity

^a^Data analyses were based on weighted estimates with sample weights provided by NHANES

^b^Mental health was assessed by a 9-item depression screening instrument, the PHQ-9. The cut-off of 5–9 and ≥ 10 was used to define the presence of mild and major depression, respectively

^c^Data are presented as weighted mean (standard error)

### Independent associations of healthy lifestyle score and mental health on mortality, and mediation analysis of mental health on associations of healthy lifestyle score with mortality among US cancer survivors

During 20,900 person-years of follow-up (median, 6.3 years [interquartile range, 3.3–10.0 years]), 819 deaths occurred, including 282 cancer and 537 non-cancer deaths. As shown in Table 2, cancer survivors with higher healthy lifestyle score had significantly decreased all-cause and non-cancer mortality. After adjusting for demographic information, sleep duration, and presence of medical conditions in model 1, and additionally adjusting for the number of cancer types and age at the first cancer diagnosis in model 2, HRs (95% CIs) for all-cause, cancer and non-cancer mortality among cancer survivors with higher healthy lifestyle score (3–5) were 0.68 (0.55–0.84), 0.69 (0.45–1.05), and 0.67 (0.51–0.88), respectively, compared to those with lower healthy lifestyle score (0–1). Each 1-point increase of healthy lifestyle score was associated with 12%, 13%, and 12% decreased risks of death from all-cause, cancer and non-cancer, respectively. These results barely changed in the fully adjusted model 3, in which PHQ-9 score was further included. Meanwhile, similar trends were observed among participants categorized by mental health, those with better mental health had significantly lower all-cause and non-cancer mortality. In the fully adjusted model 3, HRs (95% CIs) for all-cause, cancer and non-cancer mortality among cancer survivors with better mental health (PHQ-9 score = 0–4) were 0.70 (0.52–0.93), 1.02 (0.55–1.90), and 0.57 (0.40–0.80), respectively, compared to those with poor mental health (PHQ-9 score ≥ 10). Each 5-point decrease of PHQ-9 score was associated with 17%, 2%, and 24% decreased death from all-cause, cancer, and non-cancer, respectively. When higher healthy lifestyle score (3–5) was compared with lower score (0–1), the proportion mediated by the mental health was 7.8% (3.9–11.7%), 1.6% (0.8–2.4%), and 10.3% (5.2–15.3%) for all-cause, cancer, and non-cancer mortality, respectively.

**Table 2:** Independent association of healthy lifestyle score and mental health ^a^ with mortality among US cancer survivors, and mediation proportion of lifestyle inequity in mortality attributed to mental health

	Unweighted total cases/deaths	HR (95% CI)			Mediation proportion (%) (95% CI)
		Model 1^b^	Model 2^c^	Model 3 ^d^	
All-cause mortality					
Healthy lifestyle score					
0–1	1206/341	1.00 (Reference)	1.00 (Reference)	1.00 (Reference)	
2	1132/308	0.91 (0.75–1.10)	0.94 (0.78–1.13)	0.93 (0.77–1.11)	6.2 (3.1–9.3)
3–5	807/170	0.61 (0.50–0.76)	0.68 (0.55–0.84)	0.69 (0.56–0.85)	7.8 (3.9–11.7)
Per 1-point increase		0.85 (0.79–0.91)	0.88 (0.82–0.94)	0.89 (0.83–0.95)	
PHQ-9 score					
≥ 10	318/72	1.00 (Reference)	1.00 (Reference)	1.00 (Reference)	
5–9	495/140	0.85 (0.58–1.24)	0.90 (0.62–1.30)	0.92 (0.63–1.33)	
0–4	2332/607	0.61 (0.46–0.81)	0.68 (0.51–0.91)	0.70 (0.52–0.93)	
Per 5-point decrease		0.79 (0.71–0.87)	0.83 (0.74–0.92)	0.83 (0.74–0.93)	
Cancer mortality					
Healthy lifestyle score					
0–1	1206/122	1.00 (Reference)	1.00 (Reference)	1.00 (Reference)	
2	1132/106	0.91 (0.65–1.29)	0.95 (0.68–1.35)	0.95 (0.68–1.34)	1.2 (0.6–1.8)
3–5	807/54	0.63 (0.42–0.96)	0.69 (0.45–1.05)	0.69 (0.46–1.05)	1.6 (0.8–2.4)
Per 1-point increase		0.84 (0.73–0.98)	0.87 (0.76–1.00)	0.87 (0.76–1.00)	
PHQ-9 score					
≥ 10	318/29	1.00 (Reference)	1.00 (Reference)	1.00 (Reference)	
5–9	495/46	1.26 (0.70–2.27)	1.31 (0.74–2.33)	1.34 (0.75–2.40)	
0–4	2332/207	0.91 (0.51–1.63)	1.00 (0.54–1.86)	1.02 (0.55–1.90)	
Per 5-point decrease		0.94 (0.75–1.18)	0.97 (0.76–1.24)	0.98 (0.76–1.25)	
Non-cancer mortality					
Healthy lifestyle score					
0–1	1206/219	1.00 (Reference)	1.00 (Reference)	1.00 (Reference)	
2	1132/202	0.91 (0.72–1.14)	0.92 (0.74–1.14)	0.90 (0.73–1.11)	8.3 (4.1–12.2)
3–5	807/116	0.60 (0.46–0.78)	0.67 (0.51–0.88)	0.68 (0.52–0.89)	10.3 (5.2–15.3)
Per 1-point increase		0.85 (0.77–0.93)	0.88 (0.80–0.98)	0.89 (0.80–0.98)	
PHQ-9 score					
≥ 10	318/43	1.00 (Reference)	1.00 (Reference)	1.00 (Reference)	
5–9	495/94	0.67 (0.43–1.04)	0.73 (0.46–1.15)	0.74 (0.47–1.17)	
0–4	2332/400	0.49 (0.35–0.67)	0.56 (0.40–0.79)	0.57 (0.40–0.80)	
Per 5-point decrease		0.71 (0.63–0.81)	0.76 (0.67–0.85)	0.76 (0.67–0.86)	

^a^Mental health was assessed by a 9-item depression screening instrument, the Patient Health Questionnaire (PHQ-9). The cut-off of 5–9 and ≥ 10 was used to define the presence of mild and major depression, respectively

^b^Model 1 was adjusted for age at the time of interview, sex, education level, ratio of family income to poverty, race and ethnicity, sleep duration, prevalent diabetes, hypertension, dyslipidemia, history of cardiovascular disease

^c^Model 2 was additionally adjusted for the number of cancer types and age at the first cancer diagnosis

^d^Model 3 was additionally adjusted for PHQ-9 score or healthy lifestyle score, in regard to the association of healthy lifestyle score and mental health with mortality, respectively

*PHQ* Patient Health Questionnaire, *HRs* hazard ratios, *CIs* confidence interval

### Interaction and joint associations of healthy lifestyle score and mental health on mortality among US cancer survivors

**Fig. 1:**
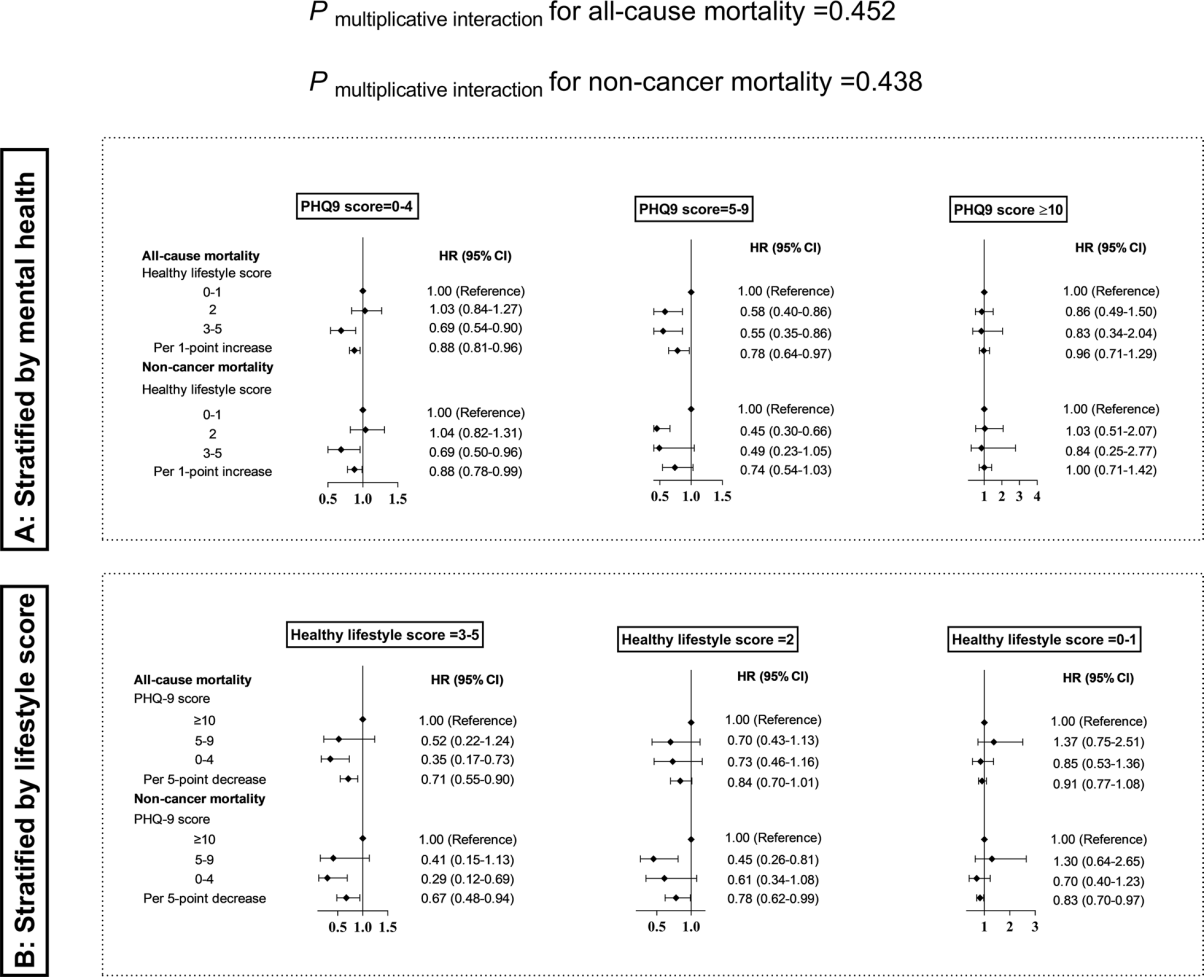
Stratified analyses. **A** Association of healthy lifestyle score with mortality among US cancer survivors stratified by mental health. **B** Association of mental health with mortality among US cancer survivors stratified by healthy lifestyle score. Mental health was assessed using the PHQ-9. Mild and major depression was defined by the cut-off of PHQ-9 score 5–9 and ≥ 10, respectively. The following covariates were adjusted: age at the time of interview, sex, education level, ratio of family income to poverty, race and ethnicity, sleep duration, prevalent diabetes, hypertension, dyslipidemia, history of cardiovascular disease, the number of cancer types, age at the first cancer diagnosis. The multiplicative interaction was evaluated by including a cross-product term of healthy lifestyle score (0–1, 2, 3–5) and mental health (PHQ-9 score 0–4, 5–9, ≥ 10) into the corresponding models. Abbreviations: PHQ-9, Patient Health Questionnaire; HR, hazard ratio; CI, confidence interval

Stratified analyses are shown in Fig. 1. The significant associations of healthy lifestyle score with all-cause and non-cancer mortality remained consistent among the cancer survivors with better mental health (PHQ-9 = 0–4), other than their counterparts with poor mental health (PHQ-9 = 5–9 or ≥ 10). Meanwhile, the significant associations of mental health with all-cause and non-cancer mortality also remained consistent among the cancer survivors with higher healthy lifestyle score (3–5), other than their counterparts with lower healthy lifestyle score (0–1). However, the multiplicative interactions of healthy lifestyle score with mental health on mortality did not reach statistical significance (*P*_multiplicative interaction_ > 0.05).

**Fig. 2:**
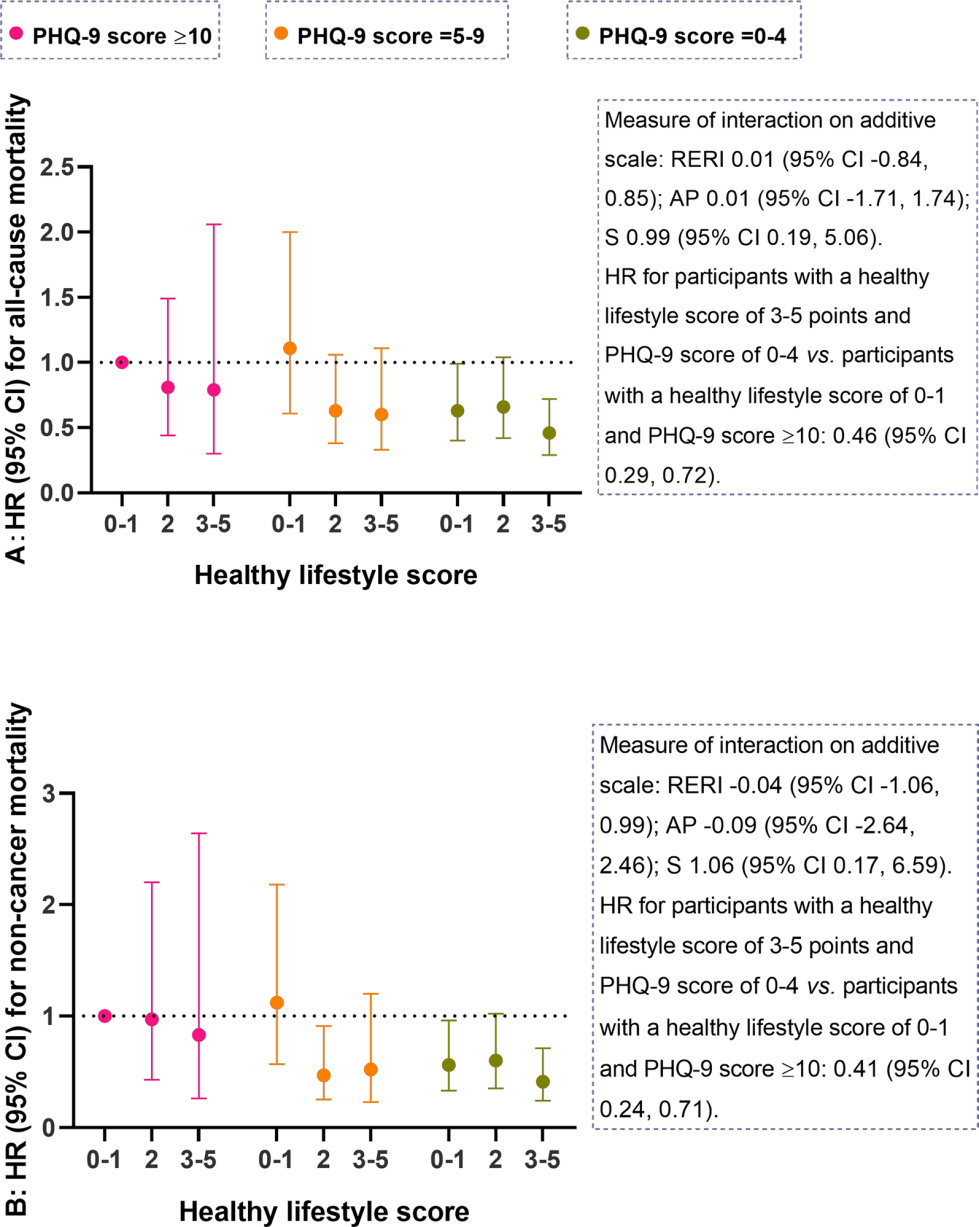
Joint associations of healthy lifestyle score and mental health on mortality among US cancer survivors. **A** HR (95% CI) for all-cause mortality. **B** HR (95% CI) for non-cancer mortality. Mental health was assessed using PHQ-9. Weighted Cox regression models were used to estimate the HR (the solid symbols) with 95% CI (the error bars) of joint categories of healthy lifestyle score and mental health for mortality. The following covariates were adjusted: age at the time of interview, sex, education level, ratio of family income to poverty, race and ethnicity, sleep duration, prevalent diabetes, hypertension, dyslipidemia, history of cardiovascular disease, the number of cancer types, age at the first cancer diagnosis. Additive interaction effects between the lifestyle score (0–1 point *vs.* 3–5 points) and mental health (PHQ-9 score ≥ 10 vs. PHQ-9 score = 0–4) were evaluated. *PHQ-9* Patient Health Questionnaire, *HR* hazard ratios, *CI* confidence interval; *RERI* relative excess risk due to interaction; *AP* attributable proportion, *S* synergy index

The joint association is shown in Fig. 2. As expected, combinations of higher healthy lifestyle score and better mental health were associated with significant decreased mortality, the lowest mortality was seen in participants with a healthy lifestyle score of 3–5 and concurrently with a PHQ-9 score of 0–4, HRs (95% CIs) was 0.46 (0.29–0.72), and 0.41 (0.24–0.71) for all-cause and non-cancer mortality, respectively. However, the additive interactions of healthy lifestyle score with mental health on mortality did not reach statistical significance (95% CIs of RERI or AP included 0, 95% CI of S included 1).

### Sensitivity analyses

After excluding participants died during the first 2-year of follow-up, or with missing covariates, the independent associations of healthy lifestyle score and mental health on mortality remained largely unchanged (Additional file 1: Table S4). Also, after reconstructing a series of new healthy lifestyle scores, the favorable associations of healthy lifestyle score with mortality were mainly consistent (Additional file 1: Table S5). After omitting 1 lifestyle from the score each time (Additional file 1: Table S6), the associations of healthy lifestyle score with mortality were all slightly changed to varying degrees, which confirmed the necessity of evaluating a combined lifestyle when exploring lifestyle and mortality outcomes, rather than just considering a single lifestyle.

## Discussion

In this cohort study of a nationally representative sample of US cancer survivors, participants with higher healthy lifestyle score had better mental health, and vice versa. Moreover, participants with higher healthy lifestyle score or better mental health had better medical conditions. During a median of 6.3 years of follow-up, cancer survivors with higher healthy lifestyle score had significantly decreased all-cause and non-cancer mortality, 6.2–10.3% of the associations were mediated by mental health. Similar trends were observed between mental health and all-cause as well as non-cancer mortality, independent of healthy lifestyle score. Results from stratified and joint analyses showed that, cancer survivors with better mental health seemed to benefit more from adopting healthy lifestyles, and vice versa. Combinations of higher healthy lifestyle score and better mental health were associated with significant decreased mortality, the lowest mortality was seen in participants with a healthy lifestyle score of 3–5 and concurrently with a PHQ-9 score of 0–4. However, neither significant multiplicative nor additive interactions of healthy lifestyle score with mental health on mortality were observed.

In 2020, Zhang and colleagues [3] summarized data from 30 studies with 1.8 million participants and concluded that participants with the healthiest combined lifestyles (including but not limited to maintaining healthy body weight, having better diet quality, staying physically active, avoiding heavy alcohol drinking and smoking) had a significant lower cancer mortality. Of note, those 30 studies were all conducted among general population. To our best knowledge, there were only seven existing studies exploring the association of combined lifestyle factors with mortality among survivors suffered from colorectal cancer [13, 39–41], breast cancer [42, 43] or pan-cancer [44]. Although consistent results were derived from these studies that greater adherence to combined healthy lifestyle was associated with improved survival among cancer survivors, several important issues should be noted. For example, smoking was not included as a component in the scores [3, 10–18], diet quality was not assessed by the comprehensive index such as HEI [3, 10–18], and the generalization was limited since those studies did not use national data. Recently, in a prospective analysis using the national data from NHANES III (1988–1994) with 522 cancer survivors, Karavasiloglou and colleagues [44] created a healthy lifestyle score based on never smoker, healthy body weight, participation in moderate to vigorous physical activity ≥ 5 times/week, moderate alcohol consumption, and high diet quality (assessed by HEI). Results showed that higher lifestyle score was associated with lower mortality (HR_3-5 score vs. 0 score_ = 0.57, 95% CI: 0.38, 0.85). Of note, over the past three decades in US, trends of lifestyle have greatly changed among the US population [45]. More evidence derived from recent data regarding healthy lifestyle and mortality among cancer survivors are warranted. In the present study using data from NHANES (2005–2018) with a larger sample size of cancer survivors, we confirmed the favorable associations of combined healthy lifestyle factors with mortality, by constructing a comprehensive lifestyle score including diet (assessed by HEI-2015), PA, smoking, drinking, and BMI. Moreover, none of the existing studies had considered sleep status as a component in the combined lifestyle score, while sleep disorder is also one the most common symptoms experienced by cancer survivors, and is reported to be associated with survival outcomes [46]. In order to make this study comparable with other studies of the same topic, in the main analyses, we did not include sleep duration as a component in the lifestyle score, instead, we considered it as a confounder and adjusted it in the multivariate models. However, we also extended a sensitivity analysis by including sleep duration as a component in the lifestyle score, the favorable associations between lifestyle score and mortality were robust.

Mental health is another important factor that may have prognostic impact on cancer. Several existing studies have explored the associations of mental health with mortality among cancer survivors, yet the findings are still inconsistent and inconclusive, due to different population with various race or ethnicity, various types of cancer, and relatively small sample sizes. Of note, a few studies [21, 22, 26, 47] evaluated the association of mental health status before cancer diagnosis with mortality among cancer survivors, other than mental health assessed after diagnosis, which might underestimate the prevalence of mental disorder [48] and its association with prognosis outcomes. In the present study, mental health was assessed after cancer diagnosis, firstly we confirmed the high prevalence of poor mental health among US cancer survivors, with nearly one in four patients reported a status of mild or major depression. Poor mental health was especially evident among cancer survivors who were first diagnosed with cancer when they were younger than 40 years old. Moreover, our results are in line with several previous studies [21, 22, 26, 49], that cancer survivors with better mental health had significantly lower mortality, each 5-point decrease of PHQ-9 score was associated with significant 17% and 24% decreased risks of death from all-cause and non-cancer, respectively.

Several biologic pathways could explain the observed associations between healthy lifestyle, better mental health, and lower mortality among cancer survivors. For example, high quality of diet could provide multiple beneficial components [50] that exert curing effects for cancer treatment. Reasonable PA can reduce blood pressure, modify dyslipidemia, and regulate glucose metabolism, these growing understanding for the benefits of PA has prompted healthcare professionals to consider the possibilities of exercise therapy in several chronic diseases, including cancers [51]. As regarding to mental health, depression and anxiety can directly influence the endocrine and immune systems [52], by suppressing the activity of NK cells and certain DNA repair enzymes [53]. Particularly, lifestyle and mental health are mutually associated and reinforced each other, mental health is viewed as a mediator between lifestyle and health outcomes. However, we lack understanding of their interaction, mediation, and joint associations with mortality among cancer survivors. To our knowledge, this is the first study to prospectively investigate these associations of combined healthy lifestyle and mental health with mortality among cancer survivors. Mediation analyses showed that 6.2–10.3% of the associations between healthy lifestyle and mortality were mediated by mental health. As expected, results from stratified and joint analyses showed that, cancer survivors with better mental health seemed to benefit more from adopting healthy lifestyles, and vice versa. Combinations of higher healthy lifestyle score and better mental health were associated with significant decreased mortality, the lowest mortality was seen in participants with a healthy lifestyle score of 3–5 and concurrently with a PHQ-9 score of 0–4. However, neither significant multiplicative nor additive interactions of healthy lifestyle score with mental health on mortality were observed.

A major strength of this study was the nationally representative sample of US cancer survivors, which made it possible for the findings to be applicated at the population level. Based on our data, on one hand, we would like to emphasize again that a large proportion of cancer survivors (24.0%) were first diagnosed with cancer when they were younger than 40 years old, which means many people need to live long-term with cancer. However, on the other hand, many of them still maintained poor lifestyle behaviors (10.1% of them were heavy drinkers, more than half were former or current smoker), or poor mental health (nearly one in four patients reported mild or major depression). The information generated from this study may give much confidence to cancer survivors that changing one’s lifestyle and/or staying mentally healthy after cancer diagnosis can improve survival. Also, comprehensive evidence derived from this study may help the healthcare providers to encourage patients to tackle multiple cost-effective modifiable risk factors for the long-term management of cancer.

Nevertheless, several limitations should also be acknowledged. First, data on lifestyle and mental health were assessed only once at baseline, which limited the possibility to capture the long-term trajectories of adherence to healthy lifestyle and mental health during the follow-up period. Although data on lifestyle and mental health were collected post-diagnosis other than pre-diagnosis, future studies with repeated measures are warranted to assess the trajectory effects. Second, apart from long-term mortality, we lacked data on recurrence and quality of life, thus we cannot assess how much cancer survivors will benefit from adopting healthy lifestyle behaviors and staying mentally healthy on these outcomes, although we did observe that participants with better lifestyle or better mental health had better sleep status and better medical conditions. Third, we lacked information on cancer stages and treatments since NHANES did not collect these data. However, to lessen the probability of reverse causation, we adjusted a wide range of potential confounding factors in the main analyses and extended several sensitivity analyses, by excluding deaths occurring during the first 2-year of follow-up period or participants with missing covariates. Fourth, in the main analyses, we constructed the healthy lifestyle score by simply summing up the number of healthy lifestyle behaviors, assuming that each individual lifestyle had equal effects on mortality, which might not be true. However, this simple calculation might be easier for the patients to understand, and more practical for the healthcare professionals to communicate in the real world. Besides, we reconstructed a series of new healthy lifestyle scores in the sensitivity analyses, and consistent results were derived. Finally, although data on specific kind of cancer was collected by asking “What kind of cancer was it”, nearly 40 kinds of cancer were reported by participants. Limited by the relatively small sample size of this study, we could not conduct this big topic among participants with a specific cancer, to clarify whether patients with different cancers can all benefit from healthy lifestyles or/and better mental health, further studies with a larger sample size of patients with certain specific cancer are warranted.

## Conclusions

In conclusion, in this cohort study with a nationally representative sample of US cancer survivors, participants with higher healthy lifestyle score had significantly decreased all-cause and non-cancer mortality, independent of mental health. Similar trends were observed between mental health and all-cause mortality as well as non-cancer mortality, independent of lifestyle. Combinations of higher healthy lifestyle score and better mental health were associated with significant decreased mortality. The information generated from this study may give much confidence to cancer survivors that changing one’s lifestyle and/or staying mentally healthy after cancer diagnosis can improve survival. Also, it may help healthcare providers to encourage patients to tackle multiple cost-effective modifiable risk factors for the long-term management of cancer.

## Supplementary Information

**Additional file 1:** **Table S1.** Components and scoring standards for Healthy Eating Index-2015. **Table S2.** Scoring standards of each lifestyle factor recoded 0–2 points. **Table S3.** Baseline characteristics of US cancer survivors according to mental health. **Table S4.** Independent association of healthy lifestyle score and mental health with mortality among US cancer survivors, after excluding participants died within 2 years of follow-up, or with missing covariates. **Table S5.** Association of several reconstructed healthy lifestyle scores with mortality among US cancer survivors. **Table S6.** Associations of different healthy lifestyle scores consisting of 4 lifestyle factors with mortality among US cancer survivors. **Figure S1.** Flow chart of participants selection for the present study.

## Data Availability

The datasets generated and/or analyzed during the current study are available at the official site of NHANES, https://www.cdc.gov/nchs/nhanes/.

## Abbreviations

AP: Attributable proportion
BMI: Body mass index
CI: Confidence interval
COVID-19: Coronavirus disease 2019
CVD: Cardiovascular disease
DA: Daily activities
DGA: Dietary Guidelines for Americans
HEI: Healthy eating index
HR: Hazard ratios
ICD-10: International Statistical Classification of Disease, Tenth Revision
LTPA: Leisure time physical activities
NHANES: National Health and Nutrition Examination Survey
PA: Physical activity
PHQ-9: Patient Health Questionnaire
RERI: Relative excess risk due to interaction
RFIP: Ratio of family income to poverty
S: Synergy index
SE: Standard error
USDA: US Department of Agriculture
WCRF/AICR: World Cancer Research Fund/American Institute for Cancer Research
