# Supplementary material for: Combined lifestyle, mental health, and mortality in US cancer survivors: a national cohort study
Source: J Transl Med. 2022 Aug 19;20:376. doi: 10.1186/s12967-022-03584-4 (PMC9389483; doi:10.1186/s12967-022-03584-4)
Supplement: Supplementary file 1 — Additional file 1: Table S1. Components and scoring standards for Healthy Eating Index-2015. Table S2. Scoring standards of each lifestyle factor recoded 0–2 points. Table S3. Baseline characteristics of US cancer survivors according to mental health. Table S4. Independent association of healthy lifestyle score and mental health with mortality among US cancer survivors, after excluding participants died within 2 years of follow-up, or with missing covariates. Table S5. Association of several reconstructed healthy lifestyle scores with mortality among US cancer survivors. Table S6. Associations of different healthy lifestyle scores consisting of 4 lifestyle factors with mortality among US cancer survivors. Figure S1. Flow chart of participants selection for the present study. [file 12967_2022_3584_MOESM1_ESM.docx]

**Supplementary Information**

**Table S1. Components and scoring standards for Healthy Eating Index-2015.**

| **Components** | **Maximum score** | **Standard for maximum score** | **Standard for 0 score** |
| --- | --- | --- | --- |
| Total fruits | 5 | ≥0.8 cup equivalents/1000 kcal | No fruit |
| Whole fruits | 5 | ≥0.4 cup equivalents/1000 kcal | No whole fruit |
| Total vegetables | 5 | ≥1.1 cup equivalents/1000 kcal | No vegetables |
| Greens and beans | 5 | ≥0.2 cup equivalents/1000 kcal | No dark green vegetables or legumes |
| Whole gains | 10 | ≥1.5 oz equivalents/1000 kcal | No whole gains |
| Dairy | 10 | ≥1.3 cup equivalents/1000 kcal | No dairy |
| Total protein foods | 5 | ≥2.5 oz equivalents/1000 kcal | No protein foods |
| Seafood and plant proteins | 5 | ≥0.8 cup equivalents/1000 kcal | No seafood or plant proteins |
| Fatty acids | 10 | (PUFAs + MUFAs)/SFAs ≥2.5 | (PUFAs + MUFAs)/SFAs ≤1.2 |
| Refined grains | 10 | ≤1.8 oz equivalents/1000 kcal | ≥4.3 oz equivalents/1000 kcal |
| Sodium | 10 | ≤1.1 gram/1000 kcal | ≥2.0 gram/1000 kcal |
| Added sugars | 10 | ≤6.5% of energy | ≥26% of energy |
| Saturated fats | 10 | ≤8% of energy | ≥16% of energy |

Abbreviations: PUFAs, polyunsaturated fatty acids; MUFAs, monounsaturated fatty acids; SFAs, saturated fatty acids.

**Table S2. Scoring standards of each lifestyle factor recoded 0-2 points.**

| **Lifestyle** | **0 score** | **1 score** | **2 score** |
| --- | --- | --- | --- |
| BMI, kg/m^2^ | ≥30.0 or <18.5 | 25.0-29.9 | 18.5-24.9 |
| Smoking | Current smoker | Former smokers/having quit <10 years | Never smoking/having quit ≥10 years |
| Alcohol drinking | Non-drinker | Heavy drinker | Low-to moderate drinker |
| HEI-2015 | T1 | T2 | T3 |
| DA, MET.hours/week | T1 | T2 | T3 |

Abbreviations: BMI, body mass index; HEI, healthy eating index; DA, daily activity; T1, the first tercile; T2, the second tercile; T3, the third tercile.

**Table S3. Baseline characteristics ^a^ of US cancer survivors according to mental health ^b^, NHANES 2005 to 2018.**

| **Characteristics** |  | **PHQ-9 score** | | | |
| --- | --- | --- | --- | --- | --- |
|  |  | **≥10** | **5-9** | **0-4** | ***P_-value_* ^d^** |
| **HEI-2015 ^c^** |  | 50.8 (0.8) | 54.6 (0.9) | 55.7 (0.3) | **<0.001** |
| **LTPA ^c^, MET.hours/week** |  | 1.2 (0.4) | 5.3 (1.5) | 6.3 (0.7) | **<0.001** |
| **Daily activity ^c^, MET.hours/week** |  | 33.8 (5.5) | 31.8 (3.7) | 31.2 (1.9) | 0.881 |
| **Alcohol drinking status, No. (%)** |  |  |  |  | 0.617 |
| Non-drinker |  | 89 (21.8) | 128 (22.0) | 605 (21.0) |  |
| Low-to moderate drinker |  | 200 (68.6) | 337 (70.2) | 1524 (68.4) |  |
| Heavy drinker |  | 29 (9.6) | 30 (7.7) | 203 (10.6) |  |
| **Smoking status, No. (%)** |  |  |  |  | **<0.001** |
| Non-smoker |  | 110 (31.7) | 210 (41.3) | 1099 (49.0) |  |
| Current smoker |  | 116 (38.5) | 89 (19.0) | 281 (12.2) |  |
| Former smoker |  | 92 (29.9) | 196 (39.7) | 952 (38.8) |  |
| **Quiting smoking ≥10 years, No. (%)** |  | 56 (12.3) | 151 (7.9) | 773 (5.7) | **<0.001** |
| **Sleep duration, hours/day** |  |  |  |  | **<0.001** |
| 6-8 hours/day, No. (%) |  | 174 (53.7) | 308 (65.3) | 1688 (74.4) |  |
| 5-5.9 or 8.1-10 hours/day, No. (%) |  | 29 (8.5) | 67 (14.6) | 327 (15.6) |  |
| <5 or >10 hours/day, No. (%) |  | 111 (37.7) | 117 (20.1) | 310 (10.0) |  |
| **Body mass index ^c^, kg/m^2^** |  | 30.6 (0.5) | 29.9 (0.4) | 28.8 (0.2) | **0.001** |
| <18.5 kg/m^2^, No. (%) |  | 5 (1.1) | 9 (2.0) | 33 (1.4) |  |
| 18.5-24.9 kg/m^2^, No. (%) |  | 70 (22.4) | 116 (24.1) | 604 (27.0) |  |
| 25.0-29.9 kg/m^2^, No. (%) |  | 91 (27.2) | 153 (28.6) | 865 (36.2) |  |
| ≥30.0 kg/m^2^, No. (%) |  | 152 (49.4) | 217 (45.3) | 830 (35.4) |  |
| **Age at interview ^c^, years** |  | 56.3 (1.1) | 62.7 (0.8) | 63.5 (0.4) | **<0.001** |
| **Gender, No. (%)** |  |  |  |  | **<0.001** |
| Male |  | 98 (31.5) | 183 (31.3) | 1211 (46.8) |  |
| Female |  | 220 (68.5) | 312 (68.7) | 1121 (53.2) |  |
| **Ethnicity, No. (%)** |  |  |  |  | **0.001** |
| Non-Hispanic white |  | 190 (78.5) | 336 (86.4) | 1619 (87.4) |  |
| Non-Hispanic black |  | 43 (7.1) | 72 (5.6) | 336 (4.8) |  |
| Mexican American |  | 38 (4.4) | 28 (2.4) | 137 (2.2) |  |
| Others |  | 47 (10.0) | 59 (5.6) | 240 (5.6) |  |
| **Education level, No. (%)** |  |  |  |  | **<0.001** |
| Less than high school |  | 103 (22.6) | 104 (13.8) | 431 (10.4) |  |
| High school or equivalent |  | 77 (27.3) | 136 (28.2) | 499 (19.3) |  |
| College or above |  | 137 (50.0) | 255 (57.9) | 1402 (70.3) |  |
| **Family income-to-poverty ratio, No. (%)** |  |  |  |  | **<0.001** |
| <1.3 |  | 146 (37.9) | 128 (18.0) | 381 (10.1) |  |
| 1.3-3.5 |  | 108 (39.7) | 212 (45.3) | 911 (35.4) |  |
| >3.5 |  | 39 (22.4) | 117 (36.7) | 857 (54.5) |  |
| **Prevalent diabetes, No. (%)** |  | 109 (26.9) | 151 (26.8) | 611 (21.3) | **0.030** |
| **Prevalent hypertension, No. (%)** |  | 211 (61.6) | 324 (61.9) | 1498 (57.6) | 0.244 |
| **Prevalent dyslipidemia, No. (%)** |  | 253 (78.6) | 379 (79.9) | 1805 (78.7) | 0.214 |
| **History of CVD, No. (%)** |  | 112 (30.3) | 138 (25.6) | 488 (16.0) | **<0.001** |
| **Number of cancer types, No. (%)** |  |  |  |  | 0.196 |
| 1 |  | 276 (86.6) | 439 (88.2) | 2105 (90.1) |  |
| 2 |  | 33 (10.7) | 48 (10.1) | 203 (8.9) |  |
| ≥3 |  | 9 (2.6) | 7 (1.8) | 23 (1.0) |  |
| **Age at cancer first diagnosed, years** |  |  |  |  | **<0.001** |
| <40 years, No. (%) |  | 103 (36.6) | 117 (28.5) | 393 (21.7) |  |
| 40-60 years, No. (%) |  | 146 (45.7) | 195 (41.0) | 908 (44.2) |  |
| >60 years, No. (%) |  | 69 (17.6) | 183 (30.5) | 1031 (34.1) |  |

Abbreviations: NHANES, National Health and Nutrition Examination Survey; PHQ, Patient Health Questionnaire; CVD, cardiovascular disease; HEI, healthy eating index; LTPA, leisure time physical activity.

^a^ Data analyses were based on weighted estimates with sample weights provided by NHANES.

^b^ Mental health was assessed by a 9-item depression screening instrument, the PHQ-9. The cut-off of 5-9 and ≥10 was used to define the presence of mild and major depression, respectively.

^c^ Data are presented as weighted mean (standard error).

**Table S4. Independent association of healthy lifestyle score and mental health ^a^ with mortality among US cancer survivors, after excluding participants died within 2 years of follow-up, or with missing covariates.**

|  | **HR (95% CI) ^b^** | | |
| --- | --- | --- | --- |
|  | **All-cause mortality** | **Cancer mortality** | **Non-cancer mortality** |
| **Excluded participants died in 2 years** |  |  |  |
| Healthy lifestyle score |  |  |  |
| 0-1 | 1.00 (Reference) | 1.00 (Reference) | 1.00 (Reference) |
| 2 | 0.93 (0.74-1.17) | 0.91 (0.61-1.35) | 0.93 (0.72-1.18) |
| 3-5 | 0.69 (0.53-0.89) | 0.61 (0.38-0.97) | 0.72 (0.53-0.97) |
| Per 1-point increase | 0.89 (0.82-0.96) | 0.84 (0.72-0.98) | 0.91 (0.82-1.01) |
| PHQ-9 score |  |  |  |
| ≥10 | 1.00 (Reference) | 1.00 (Reference) | 1.00 (Reference) |
| 5-9 | 0.71 (0.46-1.11) | 0.90 (0.43-1.88) | 0.64 (0.39-1.05) |
| 0-4 | 0.64 (0.45-0.90) | 0.83 (0.41-1.67) | 0.57 (0.39-0.84) |
| Per 5-point decrease | 0.83 (0.72-0.94) | 1.01 (0.76-1.33) | 0.76 (0.65-0.88) |
| **Excluded participants with missing covariates** |  |  |  |
| Healthy lifestyle score |  |  |  |
| 0-1 | 1.00 (Reference) | 1.00 (Reference) | 1.00 (Reference) |
| 2 | 0.91 (0.75-1.10) | 0.94 (0.66-1.35) | 0.88 (0.72-1.09) |
| 3-5 | 0.68 (0.55-0.83) | 0.69 (0.45-1.06) | 0.66 (0.51-0.86) |
| Per 1-point increase | 0.88 (0.82-0.94) | 0.87 (0.75-1.00) | 0.88 (0.79-0.97) |
| PHQ-9 score |  |  |  |
| ≥10 | 1.00 (Reference) | 1.00 (Reference) | 1.00 (Reference) |
| 5-9 | 0.90 (0.61-1.32) | 1.32 (0.72-2.41) | 0.73 (0.46-1.16) |
| 0-4 | 0.69 (0.51-0.94) | 0.97 (0.52-1.83) | 0.58 (0.41-0.83) |
| Per 5-point decrease | 0.84 (0.74-0.94) | 0.96 (0.75-1.24) | 0.77 (0.68-0.88) |

Abbreviations: HR, hazard ratio; CI, confidence interval; PHQ, Patient Health Questionnaire.

^a^ Mental health was assessed by a 9-item depression screening instrument, the Patient Health Questionnaire (PHQ-9). The cut-off of 5-9 and ≥10 was used to define the presence of mild and major depression, respectively.

^b^ Covariates adjusted: age at the time of interview, sex, education level, ratio of family income to poverty, race and ethnicity, sleep duration, prevalent diabetes, hypertension, dyslipidemia, history of cardiovascular disease, the number of cancer types, age at the first cancer diagnosis, PHQ-9 score or healthy lifestyle score was additionally adjusted in regarding to the association of healthy lifestyle score and mental health with mortality, respectively.

**Table S5. Association of several reconstructed healthy lifestyle scores with mortality among US cancer survivors.**

|  | **HR (95% CI) ^a^** | | |
| --- | --- | --- | --- |
|  | **All-cause mortality** | **Cancer mortality** | **Non-cancer mortality** |
| **Replaced LTPA with DA** | |  |  |
| Healthy lifestyle score |  |  |  |
| 0-1 | 1.00 (Reference) | 1.00 (Reference) | 1.00 (Reference) |
| 2 | 0.82 (0.68-1.00) | 0.82 (0.62-1.08) | 0.81 (0.64-1.04) |
| 3-5 | 0.78 (0.64-0.95) | 0.85 (0.60-1.18) | 0.74 (0.56-0.97) |
| Per 1-point increase | 0.89 (0.83-0.96) | 0.90 (0.79-1.02) | 0.89 (0.80-0.99) |
| **Included sleep duration into score** | |  |  |
| Healthy lifestyle score ^b^ |  |  |  |
| 0-2 | 1.00 (Reference) | 1.00 (Reference) | 1.00 (Reference) |
| 3 | 0.89 (0.76-1.05) | 0.91 (0.67-1.23) | 0.87 (0.71-1.07) |
| 4-6 | 0.61 (0.49-0.76) | 0.65 (0.41-1.02) | 0.57 (0.44-0.75) |
| Per 1-point increase | 0.87 (0.82-0.92) | 0.86 (0.76-0.97) | 0.87 (0.80-0.95) |
| **Each lifestyle coded 0-2 points** | |  |  |
| Healthy lifestyle score |  |  |  |
| 0-4 | 1.00 (Reference) | 1.00 (Reference) | 1.00 (Reference) |
| 5-7 | 0.84 (0.69-1.03) | 0.88 (0.64-1.21) | 0.81 (0.63-1.05) |
| 8-10 | 0.69 (0.52-0.91) | 0.72 (0.45-1.14) | 0.67 (0.48-0.92) |
| Per 1-point increase | 0.90 (0.86-0.95) | 0.91 (0.84-0.99) | 0.89 (0.84-0.95) |
| **Weighted lifestyle score** |  |  |  |
| Healthy lifestyle score |  |  |  |
| T1 | 1.00 (Reference) | 1.00 (Reference) | 1.00 (Reference) |
| T2 | 0.86 (0.68-1.09) | 0.89 (0.64-1.23) | 0.85 (0.65-1.09) |
| T3 | 0.61 (0.48-0.78) | 0.60 (0.39-0.93) | 0.61 (0.44-0.85) |

Abbreviations: HR, hazard ratio; CI, confidence interval; LTPA, leisure time physical activity; DA, daily activity; T1, the first tercile; T2, the second tercile; T3, the third tercile.

^a^ Covariates adjusted: age at the time of interview, sex, education level, ratio of family income to poverty, race and ethnicity, sleep duration, prevalent diabetes, hypertension, dyslipidemia, history of cardiovascular disease, the number of cancer types, age at the first cancer diagnosis, PHQ-9 score.

^b^ Sleep duration was not adjusted.

**Table S6.** **Associations of different healthy lifestyle scores consisting of 4 lifestyle factors with mortality among US cancer survivors.**

|  | **Healthy lifestyle score, HR (95% CI) ^a^** | | | |
| --- | --- | --- | --- | --- |
|  | 0-1 | 2 | 3-4 | Per 1-point increase |
| **Omit LTPA** |  |  |  |  |
| All-cause mortality | 1.00 (Reference) | 0.93 (0.77-1.11) | 0.77 (0.61-0.98) | 0.92 (0.85-0.99) |
| Cancer mortality | 1.00 (Reference) | 1.02 (0.74-1.41) | 0.76 (0.47-1.24) | 0.90 (0.77-1.05) |
| Non-cancer mortality | 1.00 (Reference) | 0.87 (0.71-1.07) | 0.76 (0.56-1.03) | 0.92 (0.82-1.02) |
| **Omit diet** |  |  |  |  |
| All-cause mortality | 1.00 (Reference) | 0.91 (0.76-1.10) | 0.69 (0.52-0.92) | 0.91 (0.83-0.99) |
| Cancer mortality | 1.00 (Reference) | 0.84 (0.59-1.21) | 0.64 (0.36-1.14) | 0.89 (0.75-1.05) |
| Non-cancer mortality | 1.00 (Reference) | 0.95 (0.76-1.18) | 0.70 (0.46-1.04) | 0.91 (0.80-1.04) |
| **Omit smoking** |  |  |  |  |
| All-cause mortality | 1.00 (Reference) | 0.91 (0.77-1.09) | 0.86 (0.68-1.09) | 0.93 (0.86-1.01) |
| Cancer mortality | 1.00 (Reference) | 0.80 (0.57-1.12) | 0.97 (0.63-1.49) | 0.92 (0.76-1.11) |
| Non-cancer mortality | 1.00 (Reference) | 0.98 (0.78-1.23) | 0.80 (0.58-1.11) | 0.93 (0.84-1.04) |
| **Omit alcohol drinking** |  |  |  |  |
| All-cause mortality | 1.00 (Reference) | 0.83 (0.67-1.02) | 0.58 (0.45-0.76) | 0.88 (0.81-0.96) |
| Cancer mortality | 1.00 (Reference) | 1.04 (0.77-1.39) | 0.54 (0.31-0.94) | 0.88 (0.76-1.01) |
| Non-cancer mortality | 1.00 (Reference) | 0.71 (0.56-0.91) | 0.58 (0.41-0.83) | 0.88 (0.78-0.99) |
| **Omit BMI** |  |  |  |  |
| All-cause mortality | 1.00 (Reference) | 0.81 (0.67-0.97) | 0.48 (0.37-0.61) | 0.80 (0.73-0.87) |
| Cancer mortality | 1.00 (Reference) | 0.82 (0.59-1.14) | 0.47 (0.27-0.84) | 0.78 (0.66-0.93) |
| Non-cancer mortality | 1.00 (Reference) | 0.79 (0.63-0.99) | 0.48 (0.33-0.69) | 0.80 (0.71-0.90) |

Abbreviations: HR, hazard ratio; CI, confidence interval; LTPA, leisure time physical activity; BMI, body mass index.

^a^ Covariates adjusted: age at the time of interview, sex, education level, ratio of family income to poverty, race and ethnicity, sleep duration, prevalent diabetes, hypertension, dyslipidemia, history of cardiovascular disease, the number of cancer types, age at the first cancer diagnosis, and PHQ-9 score. In addition, the omitted lifestyle factor was also included in the corresponding models.

Participants from NHANES 2005-2018 (n=70190)

Participants ≥18 years

(n=42143)

Participants without pregnancy

(n=41406)

Adult cancer survivors (n=3773)

Eligible adult cancer survivors

(n=3145)

Excluded if age<18 years (n=28047)

Excluded if pregnant (n=737)

Excluded if reported no history of cancer

(n=37633)

Excluded if with missing data for lifestyle factors, mental health, or mortality (n=628)

**Figure S1.** Flow chart of participants selection for the present study. Abbreviations: NHANES, National Health and Nutrition Examination Survey.
